# *TORO Indexer*: a *PyTorch*-based indexing algorithm for kilohertz serial crystallography

**DOI:** 10.1107/S1600576724003182

**Published:** 2024-06-18

**Authors:** Piero Gasparotto, Luis Barba, Hans-Christian Stadler, Greta Assmann, Henrique Mendonça, Alun W. Ashton, Markus Janousch, Filip Leonarski, Benjamín Béjar

**Affiliations:** a Scientific Computing Division, Paul Scherrer Institute, Villigen, Switzerland; b Swiss Data Science Center, Paul Scherrer Institute, Villigen, Switzerland; c Swiss Light Source, Paul Scherrer Institute, Villigen, Switzerland; d Swiss National Supercomputing Centre, Lugano, Switzerland; Uppsala University, Sweden; The European Extreme Light Infrastructure, Czechia

**Keywords:** *PyTorch* indexer, robust optimization, real-time indexing algorithms, serial crystallography, macromolecular crystallography, X-ray image acquisition, *Torch* scripts

## Abstract

Serial crystallography (SX) involves combining observations from a very large number of diffraction patterns coming from crystals in random orientations. To compile a complete data set, these patterns must be indexed (*i.e.* their orientation determined), integrated and merged. Introduced here is *TORO* (*Torch*-powered robust optimization) *Indexer*, a robust and adaptable indexing algorithm developed using the *PyTorch* framework. *TORO* is capable of operating on graphics processing units (GPUs), central processing units (CPUs) and other hardware accelerators supported by *PyTorch*, ensuring compatibility with a wide variety of computational setups. In tests, *TORO* outpaces existing solutions, indexing thousands of frames per second when running on GPUs, which positions it as an attractive candidate to produce real-time indexing and user feedback. The algorithm streamlines some of the ideas introduced by previous indexers like *DIALS* real-space grid search [Gildea, Waterman, Parkhurst, Axford, Sutton, Stuart, Sauter, Evans & Winter (2014). *Acta Cryst.* D**70**, 2652–2666] and *XGandalf* [Gevorkov, Yefanov, Barty, White, Mariani, Brehm, Tolstikova, Grigat & Chapman (2019). *Acta Cryst.* A**75**, 694–704] and refines them using faster and principled robust optimization techniques which result in a concise code base consisting of less than 500 lines. On the basis of evaluations across four proteins, *TORO* consistently matches, and in certain instances outperforms, established algorithms such as *XGandalf* and *MOSFLM* [Powell (1999). *Acta Cryst.* D**55**, 1690–1695], occasionally amplifying the quality of the consolidated data while achieving superior indexing speed. The inherent modularity of *TORO* and the versatility of *PyTorch* code bases facilitate its deployment into a wide array of architectures, software platforms and bespoke applications, highlighting its prospective significance in SX.

## Abstract 2

Serial crystallography (SX) requires efficient processing of numerous diffraction patterns. *TORO Indexer* is a high-performance indexing solution that operates across various platforms such as GPUs, CPUs and TPUs, offering high processing speed without compromising indexing quality. Its design ensures easy integration into existing software, making it a useful tool for evolving SX techniques with ever-expanding data volumes.

## 1. Introduction

**Figure 1:**
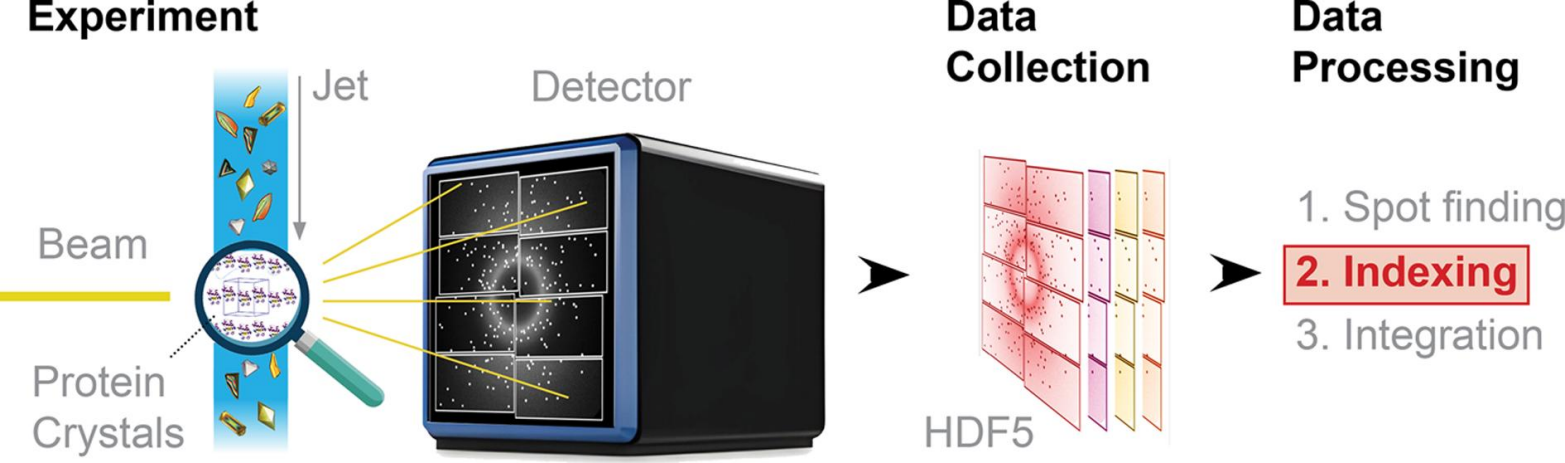
Schematic representation of an example serial crystallography experiment. The X-ray beam illuminates a liquid jet, *i.e.* a medium loaded with protein crystals. Upon beam–crystal interaction, a diffraction image is captured by the detector. Advancements in detector technology enable data collection rates exceeding 1 kHz, resulting in vast data sets (typically several terabytes). Primary data processing involves identifying images with distinct signals and pinpointing strong reflections through spot finding. Subsequent indexing associates spots with the corresponding Miller indices, gathering the requisite statistics for integration and merging.

Serial crystallography (SX) is a technique with applications in the field of structural biology (Chapman *et al.*, 2011 ▸; Stellato *et al.*, 2014 ▸) and chemistry (Higashino *et al.*, 2023 ▸; Schriber *et al.*, 2022 ▸). Unlike traditional crystallography, which collects diffraction from one single large crystal from multiple (fixed) viewing angles, SX collects a series of diffraction patterns coming from thousands or even millions of randomly oriented micro- and nano-crystals, for example using a high-viscosity extrusion jet (Grünbein & Nass Kovacs, 2019 ▸) (see Fig. 1 ▸), and merges them to determine the three-dimensional atomic structure of macromolecules.

SX is especially valuable for studying molecules that do not readily form large high-quality crystals, as well as for capturing ultrafast (femto- to millisecond) dynamic processes through time-resolved experiments (Weinert *et al.*, 2019 ▸; Wranik *et al.*, 2023 ▸). SX experiments are usually performed at X-ray free electron lasers (Boutet *et al.*, 2012 ▸) and bright synchrotron facilities (Leonarski *et al.*, 2023*b* ▸; Diederichs & Wang, 2017 ▸), though it is also possible to perform the experiments with electron beams (Hogan-Lamarre *et al.*, 2024 ▸).

Obtaining the final structure from the randomly oriented diffraction patterns requires indexing each diffraction pattern and merging the intensities into a complete data set, which can then be used to solve the structure. To date, several automated indexing algorithms such as *XGandalf* (Gevorkov *et al.*, 2019 ▸), *DIALS* real-space grid search (Gildea *et al.*, 2014 ▸), *MOSFLM* (Powell, 1999 ▸), *IDXREF* in *XDS* (Kabsch, 2010 ▸), *DirAx* (Duisenberg, 1992 ▸), *Pinkindexer* (Gevorkov *et al.*, 2020 ▸) and many others (Li *et al.*, 2019 ▸) are used in well established data processing suites such as *CrystFEL* (White *et al.*, 2012 ▸) and *DIALS* (Winter *et al.*, 2018 ▸).

A fundamental challenge of SX comes from the fact that the method generates massive data sets, comprising thousands to millions of diffraction patterns collected with large-format kilohertz pixel-array detectors, like for example EIGER (Förster *et al.*, 2019 ▸), JUNGFRAU (Leonarski *et al.*, 2018 ▸), CITIUS (Takahashi *et al.*, 2023 ▸) or AGIPD (Gisriel *et al.*, 2019 ▸). While the advancements in detector technology allow the collection of complete data sets in a shorter time, very high data volumes become a significant challenge for the computing infrastructure (Leonarski *et al.*, 2020 ▸).

To deal with this challenge, we introduce the *TORO* (*Torch*-powered robust optimization) *Indexer* algorithm, which in the current implementation is applicable when the unit-cell parameters are known. *TORO* features:

(i) High-speed (≥2 kHz) indexing of serial crystallography data.

(ii) State-of-the-art indexing quality.

(iii) A user-friendly interface, thanks to its *PyTorch*-based design for prototyping and integration into Python pipelines with C++ deployment capabilities.

(iv) A seamless variable computational setup for running on different architectures, *e.g.* graphics, tensor and central processing units (GPUs, TPUs and CPUs, respectively).

To achieve real-time data processing, software usually undergoes a complex series of optimizations, aimed at fully utilizing the available hardware. Significant gains can be achieved through use of hardware accelerators (Thompson & Spanuth, 2021 ▸; Peccerillo *et al.*, 2022 ▸), like for example GPUs, field-programmable gate arrays (FPGAs) and TPUs. When these accelerators were first introduced in a general-purpose computing context, programming them required low-level frameworks, like CUDA (Nickolls *et al.*, 2008 ▸) for GPUs or register transfer languages for FPGAs. This is a lengthy process that requires specific knowledge and expertise.

With the advent of modern machine learning (ML) frameworks like *PyTorch* (Paszke *et al.*, 2019 ▸) and *TensorFlow* (https://www.tensorflow.org/), the landscape of software development is witnessing a transformation. Designed for modularity and efficiency, these frameworks empower a wider audience of researchers to translate intricate ideas, particularly those centred on tensorial operations, into functional solutions rapidly with a minimal code footprint. This marriage of tensor computing capabilities in advanced ML frameworks with scientific computing is what made *TORO* possible. While no machine learning is involved in our algorithm, these frameworks remain a powerhouse when dealing with optimization and linear algebra operations, tools we thoroughly exploit in the implementation of our indexer. To draw a comparison, the indexing algorithm *XGandalf*, primarily crafted in C++, involves an extensive code base (several thousand lines) and is limited to CPU processing. In contrast, *TORO*, developed using *PyTorch*, is encapsulated within less than 500 lines of high-level Python code. The difference is mostly in the fact that low-level code has to incorporate a lot of ‘infrastructure’ code, which includes details of how to implement the operations on a CPU or other device. On the other hand, *PyTorch* code contains only a description of the mathematical computations. This makes it easy for scientists to understand the algorithm from the code and adapt *TORO* for applications not covered within this paper. Moreover, the C++ back end of *PyTorch* allows for seamless deployment in both C++ and Python projects.

Despite its concise code base, *TORO* delivers indexing quality on a par with *XGandalf* yet is versatile enough to operate on GPUs, TPUs and CPUs alike. With the change of a single line of code, *TORO* is optimized to run on modern GPUs. This allows for parallel indexing of spot patterns within large batches, leading to indexing performance that surpasses the 2 kHz regime requirement of modern detectors like JUNGFRAU (4M) (Leonarski *et al.*, 2018 ▸). As with most indexing algorithms, *TORO* offers a trade-off between speed and quality by choosing different hyper­parameters. However, even in the fastest setting we studied, the drop in indexing quality is barely noticeable.

*TORO* also profits from optimization methods readily available in modern ML frameworks. By employing robust optimization methods resilient to outliers and closed-form updates at each algorithmic step, we can reject outliers in a principled way and achieve fast convergence of the estimates in a small number of iterations (see Section 2.1.1 for details). This gives *TORO* a competitive advantage in terms of robustness and efficiency over existing methods (Gevorkov *et al.*, 2019 ▸; Winter *et al.*, 2018 ▸) that employ gradient-descent updates for refinement of the estimates and heuristic rules for outlier removal.

In the following sections, we introduce the problem formally and then proceed to describe our algorithm and put it in context by comparing it with other indexers. Section 3 presents the results of both the indexing quality and computational performance of *TORO*. We show that *TORO* achieves state-of-the-art indexing quality by comparing it with *XGandalf* and *MOSFLM* on different protein data sets. We then turn to computational performance. Our benchmarks show over a 1000 times speed-up with *TORO* over *XGandalf* on a fixed curated data set, designed to be representative of the needs of SX.

## 2. Methods

### 2.1. The indexing problem

Indexing involves identifying Bragg spots (reflections) observed in frames containing a diffraction pattern and using them to infer the crystal orientation. The process begins by mapping the positions of Bragg spots on the detector to a set of vectors in three-dimensional reciprocal space lying on the Ewald sphere (Drenth, 2007 ▸). Such mapping can be computed on the basis of the experimental setup (*i.e.* detector geometry, wavelength and incident direction of the beam *etc.*). The resulting set of points in three-dimensional reciprocal space, denoted 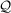, is a subset of a rotated version of the reciprocal lattice which is initially unknown. We refer to the points in 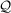 as ‘reciprocal spots’. We formalize the mathematical problem of indexing a set of points 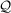 in reciprocal space.

The following expression describes the Laue equation. Let 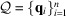, 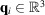, be a set of *n* points in reciprocal space and assume that there is a subset 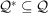 and three vectors 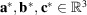 being the crystal lattice basis vectors such that for each 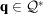 there exist integers 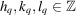 such that the following (ideal) Laue condition is satisfied: 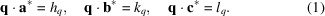
We refer to *h*_*q*_, *k*_*q*_, *l*_*q*_ as the Miller indices of 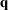. That is, there is a subset of the integer grid 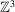 that gets mapped to 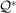 using the matrix 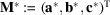. In practice, real data are intrinsically noisy and thus we aim to satisfy the Laue condition up to some bounded error, *i.e.* we assume that there is a known *maximum absolute allowed error* which is a hyperparameter of our algorithm.

Formally, the indexing problem consists of determining 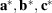 and 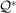 using solely 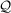 as an input. This problem is challenging as 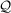 may contain outlier points due to noise and false detections that do not satisfy the Laue condition, *i.e.* they could be arbitrary points. We also assume that, in each problem, the ideal properties of the crystal lattice basis vectors are known, *i.e.* we know the reference norm of 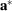, 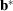 and 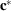, as well as the angles between them, that any valid solution should approximate. That is, we can assume that we are given a matrix 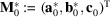, being the given *ideal* crystal lattice basis with some arbitrary orientation.

We say that a basis matrix 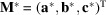 is a solution to the indexing problem if, for a subset 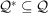 with at least *k*_min_ elements, it holds that 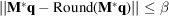 for all 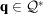, where β is the bound on the maximum allowed absolute error mentioned above. That is, we have a basis 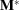 for which all reciprocal spots in 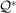 satisfy the Laue condition up to the maximum allowed error. We aim to have solutions that resemble the structure of the given ideal crystal lattice basis 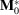, so if 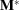 differs much from this structure we do not consider it as a valid solution.

#### 2.1.1. *TORO*: algorithm description

Indexing algorithms usually operate in two phases. Initially, they attempt to identify potential candidates for the vectors 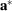, 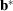 and 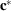. In the subsequent phase, these candidates are assembled to form a solution that exhibits a similar structure to the given matrix 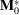. A final tuning phase can be employed to refine the solutions.

Our algorithm follows a similar two-phase approach, but we emphasize the differences, in particular with *XGandalf*, as follows. In *XGandalf*, the first phase involves sampling vectors and optimizing them using gradient descent (GD). However, GD is inherently sequential, which hinders parallelism and, in the case of *XGandalf*, requires a large number of iterations and employs a set of hand-crafted functions to avoid being trapped in local minima. The final tuning phase also relies on GD. Furthermore, using GD is not inherently robust, as it does not effectively account for outliers; instead, *XGandalf* combines several heuristics to mitigate their influence. *TORO* uses robust optimization for outlier removal instead, as described in Section 2.1.2 below.

Another issue with the approach of *XGandalf* is that candidate vectors for 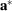, 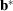 and 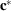 are only optimized independently and then a solution basis is put together from these candidate vectors. That is, in order to index correctly, the algorithm requires all three vectors of the solution to be present in the candidate vectors, which decreases the chances of finding a solution. Moreover, joint optimization occurs only during the final refinement step. This becomes problematic, particularly in the presence of a large number of outliers, as each candidate vector for 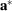, 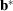 and 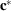 might propose a different set of outliers, leading to incompatible solutions.

In its first phase, *TORO* also involves a sampling strategy to obtain candidates for the vectors 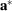, 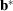 and 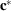, but to address the above-mentioned issues, we use a simplification of the objective function which allows us to avoid employing GD. This simpler objective has closed-form updates that accelerate computation and eliminate GD-related problems such as being stuck in local minima, zigzagging, choosing the learning rate, and gradient explosion or vanishing, among others. In fact, with no outliers, our approach only requires a single update step to reach the solution. However, handling outliers is fundamental to solving the indexing problem. Consequently, we introduce a robust optimization technique akin to the least trimmed squares (LTS) method (Víšek, 2006 ▸). This technique introduces a slight constant overhead on the number of updates compared with the regular least-squares (LS) method. However, it enables us to robustly handle any number of outliers without incurring the iterative demands often associated with GD methods.

To optimize candidate vectors jointly, in the second phase of our algorithm we utilize the candidate vectors obtained earlier to construct copies of the given basis 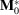 attached to these candidates. Essentially, for each candidate vector 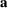 we create a copy of 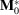 and rotate it in three dimensions so that one of its vectors aligns with 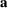. We then spin this copy around 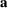 to produce more basis samples. This can be viewed as a second sampling strategy, but this time we sample entire basis matrices rather than individual vectors. While sampling directly in this matrix space without using candidate vectors seems appealing, we found it to be too computationally expensive, hence the need for a more precise two-phase sampling approach. After sampling, we apply the same robust estimation procedure mentioned earlier to simplify the objective function and perform a joint robust optimization to find the three vectors 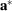, 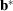 and 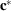. This strategy has the advantage that only a single vector from the solution needs to be present in the candidate vectors found in the first phase.

**Figure 2:**
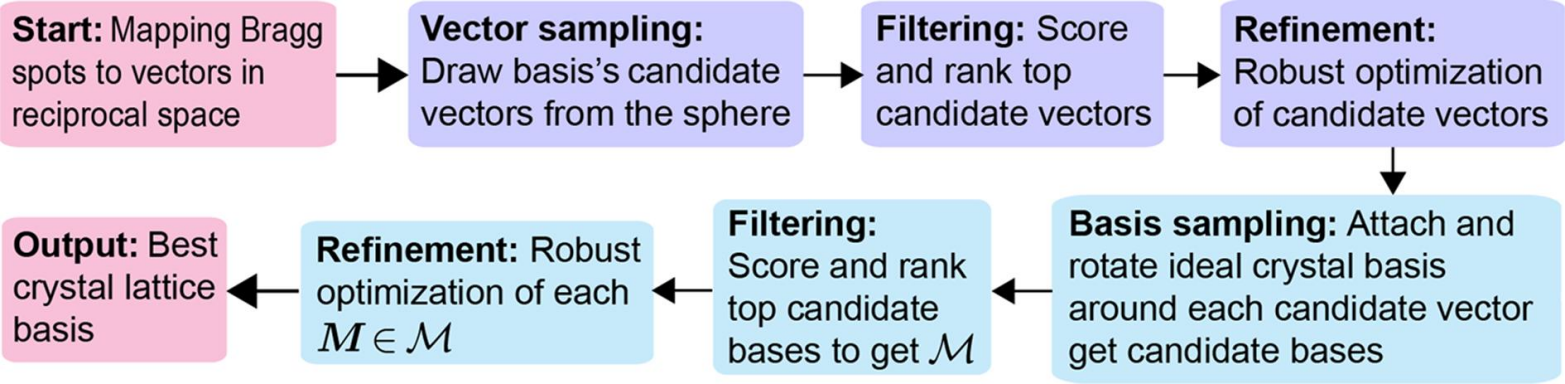
*TORO Indexer* algorithmic flow. The process starts with mapping the positions of Bragg spots to a set of vectors in three-dimensional reciprocal space. This data set is then subjected to robust optimization methods, such as least trimmed squares and residual threshold annealing, in order to identify the crystal orientation.

Fig. 2 ▸ provides an overview of the algorithm, where two score-and-rank steps are also included. These steps make use of a relaxation of the Laue condition. That is, we rank higher the candidate vectors (or matrices) that are close to satisfying the Laue condition, *i.e.* they are close enough to integers after taking the product. This allows us to focus our computational power on those that are more promising. *XGandalf* uses similar steps where only the top candidates are passed to subsequent phases.

Since each frame can be treated independently, both *XGandalf* and *TORO* are trivially parallelizable. However, due to the reduced number of iterations in *TORO* and the similarity between the computations done on different frames, we can harness the power of GPUs and TPUs, while retaining the flexibility of using multi-core CPUs when other accelerators are unavailable. Regardless of the architecture choice, *TORO* offers a faster alternative.

#### 2.1.2. Robust optimization

In this section, we explain the proposed estimation method which does not rely on using GD. Let us first consider a simplification of the problem (which we will remove in the next section). Assume that an oracle provides the target Miller indices for the reciprocal spots in 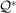, *i.e.* for each 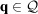 we are given 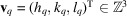 such that for each 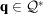

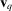
 coincides with the Miller indices of 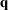 (the oracle can give us anything as 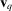 for the spots in 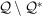 and we cannot differentiate between them *a priori*). While this is a seemingly strong assumption, we discuss later how a sampling strategy can simulate such an oracle.

Our problem then becomes that of finding 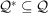 and the vectors 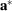, 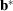 and 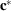 so that the Laue condition is almost satisfied with the Miller indices provided by the oracle.

Once the set 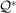 has been determined, we would like to find the basis vectors 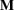 that best fit the data in the least-squares sense: 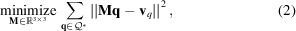
which is a simplification of the general indexing problem where the provided Miller indices 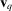 serve as discrete targets for the optimization. In addition, for a solution to be accepted, we would like the norm of all residuals 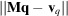, 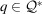, to be less than the bound β on the maximum allowed absolute error. Note that finding the optimal basis 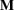 in (2) can be done in closed form, and from the obtained estimate it is straightforward to check whether the acceptance condition 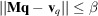, 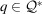, is met.

However, the subset of points 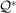 is unknown *a priori* and needs to be estimated as well. For this purpose, we propose a robust estimation method akin to LTS (Víšek, 2006 ▸). Recall that in LTS, the goal is to find a subset of a given cardinality and regression coefficients that minimize the squared error. In our case, the cardinality of the set 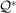 is variable as it depends on the prescribed error tolerance β. It can thus be understood as the dual version of the LTS and can then be solved as a sequence of LTS problems where the cardinality of the set is monotonically updated. The process roughly consists of the following steps:

(i) *Fitting phase.* The process starts by fitting the linear model to the current estimate of the subset 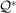.

(ii) *Residual calculation and sorting.* After fitting the model, the residuals (*i.e.*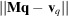) are calculated for each data point and sorted in ascending order.

(iii) *Trimming phase.* The current subset of valid points is updated by discarding all data points with large residuals.

(iv) *Refitting.* The model is then refitted using the new subset of points.

This process is repeated until the residuals go below a given threshold. More precisely, we proceed as follows:

(v) *Residual threshold annealing.* To find the solution to (2), we proceed by rounds consisting of a fitting and a trimming phase as described above. We begin by letting 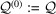, but this subset is updated in each round. In the fitting phase of the *k*th round, we minimize over 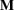 the loss in (2) by fixing 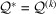. Note that such minimization is an ordinary least-squares problem, which has a closed-form solution. Once its optimum 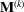 is computed, the trimming phase consists of looking at each residual, *i.e.* each 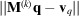, and defining 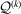 as the set with all reciprocal spots with a residual smaller than the current residual threshold. This residual threshold starts with a large enough value and is decreased monotonically at each iteration as in the following algorithm, approaching the maximum allowed error that a valid solution can have as the iterations increase.
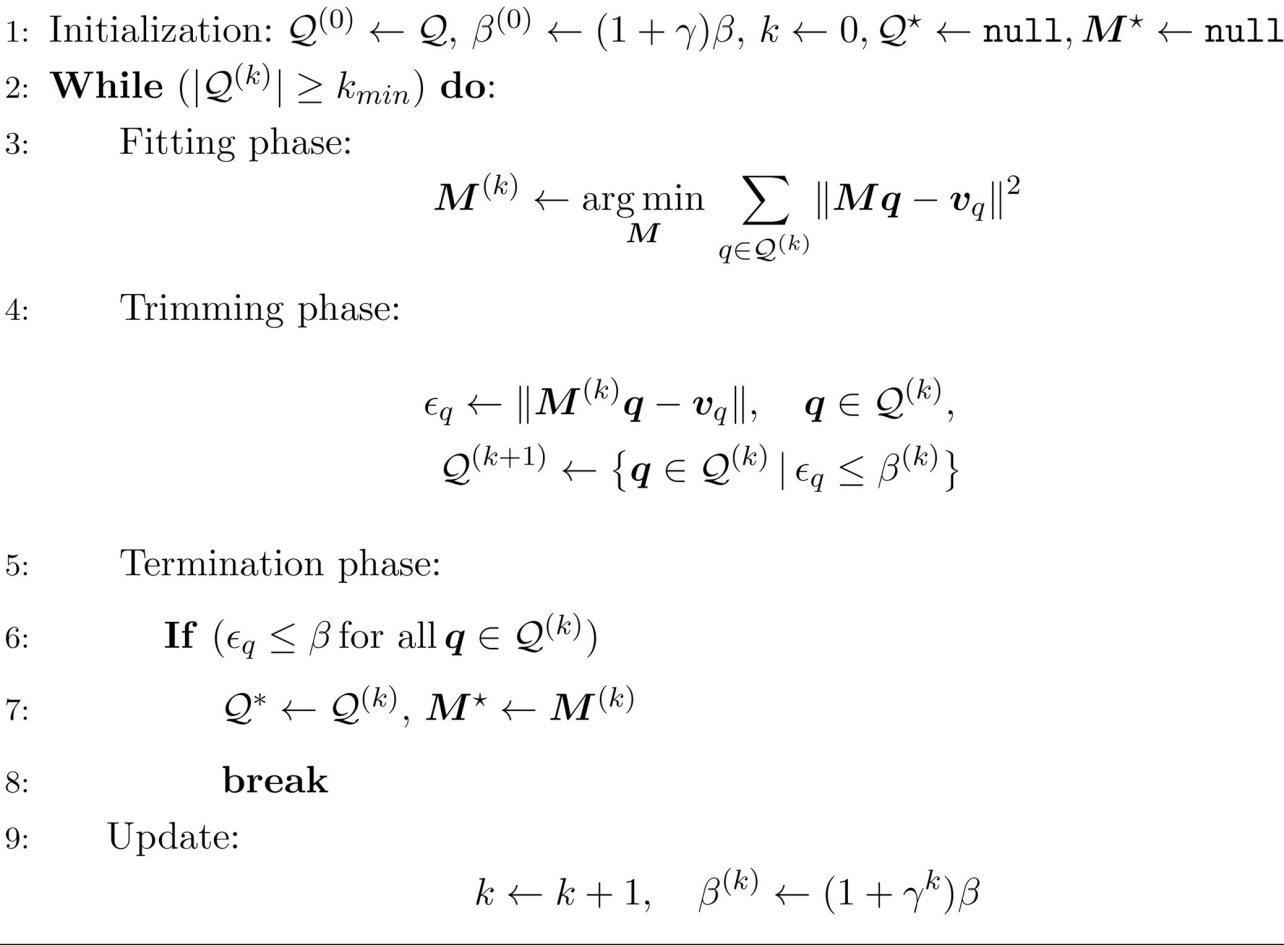


In this way, we monotonically reduce the value of the loss in (2) in each iteration. We stop whenever we go below the maximum acceptable error value and output the remaining reciprocal spots as our solution for 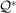, along with the corresponding 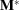. However, since we have the constraint that any valid solution should have a minimum number of reciprocal spots *k*_min_, whenever we are left with fewer than *k*_min_ reciprocal spots we stop and give the output that there is no valid solution for this instance.

A summary of the robust estimation procedure can be found in the algorithm above.

#### 2.1.3. Sampling can replace the oracle

In this section we show that, with enough computing power, a sampling strategy can play the role of the oracle invoked in the previous section. Recall that we assume that the structure of the ideal crystal lattice basis vectors is given, *i.e.* we know the ideal norms (lengths) of 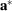, 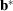, 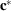 and the ideal angles between them (the actual solution might differ slightly from these ideal conditions).

If we are given an arbitrary rotation of 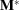, say 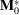, it induces a set of *possible* Miller indices for the reciprocal spots of 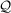 by considering the set 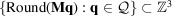. These induced Miller indices will be used to replace the oracle from the previous section as follows.

Recall that for each 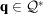, 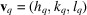 denotes the true Miller indices of 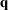. The goal of our sampling strategy is to construct candidate bases, being rotations of 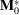, such that for at least one of them the induced Miller indices of this basis mostly coincide with the true Miller indices of 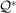. That is, we want to sample a basis 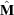 such that 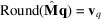 for most reciprocal spots 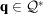. If that were the case, then these induced Miller indices would play the role of the oracle and, by running the proposed robust estimation procedure on them, the optimization will succeed in solving the indexing problem. The larger the number of candidate samples we take, the higher the chances of obtaining such 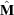 but the higher the computational cost.

**Figure 3:**
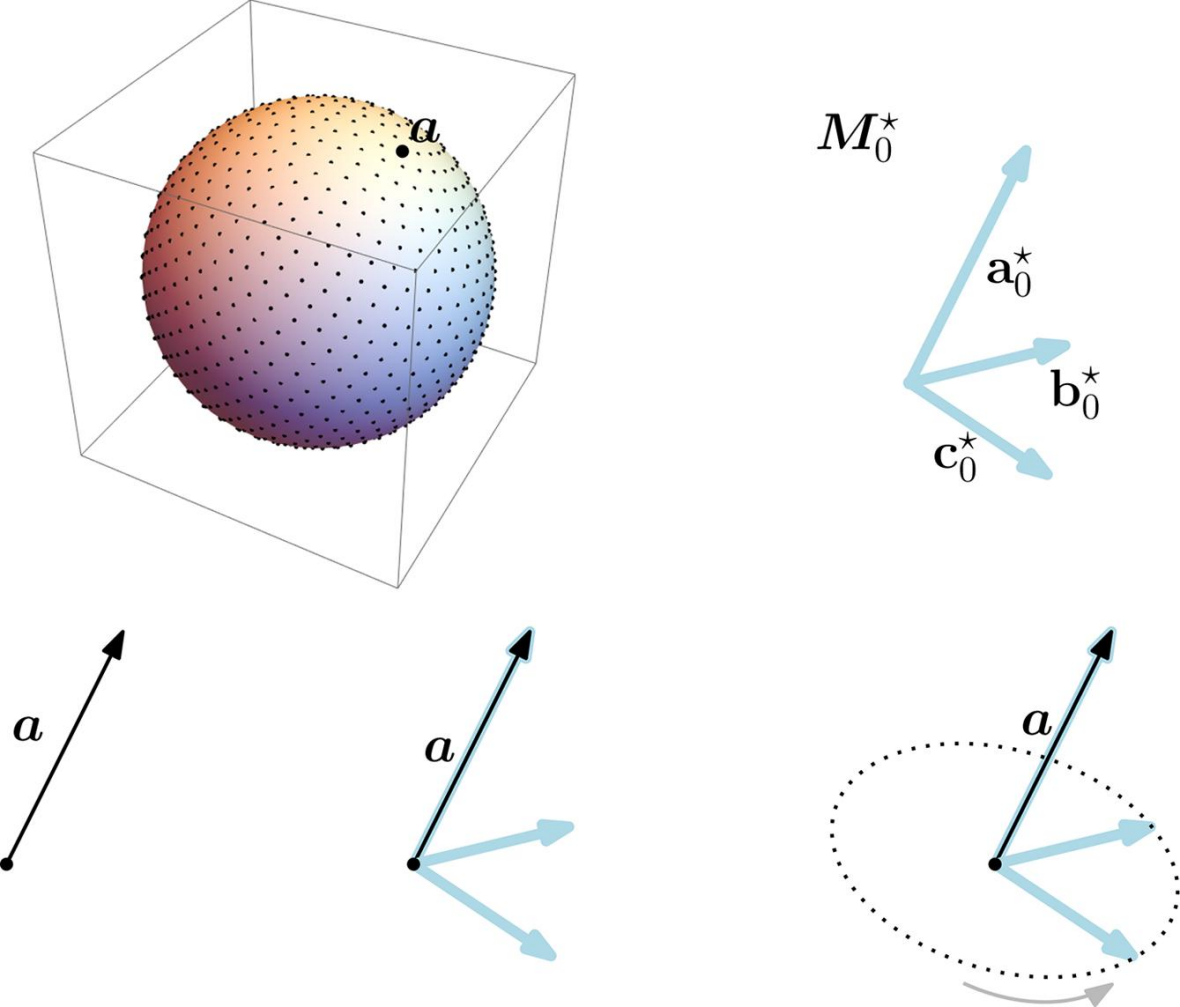
A sketch of our sampling algorithm. Points are sampled for a sphere of radius 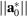. For each top-ranked sample 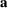 among them, a copy of 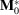 is attached to it by making 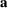 and 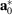 coincide. Then 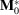 is rotated around the axis defined by 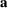. Snapshots are stored every predefined number of degrees, producing a set of rotated copies of 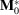 being the basis samples that correspond to 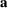.

Our sampling algorithm is described in detail in the supporting information and sketched hereafter (see also Fig. 3 ▸). We start by computing evenly spaced vectors from the surface of a sphere to be candidates for the crystal lattice basis vector 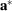 (the process is repeated in parallel for 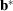 and 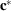, but we describe it only for 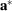 for simplicity). We rank them and keep only the most promising candidates among them. Then for each candidate 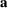, we attach a copy of the given 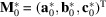 by making 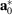 and 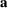 coincide. We produce several copies by spinning these bases around their fixed vector 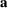. We rank these produced bases and choose the most promising among them to obtain a set 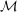 of candidate bases. Both ranking strategies prefer candidates where the Laue condition is closer to being satisfied for more reciprocal spots. Finally, for each 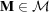, we consider the Miller indices induced by 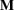 as a target for solving (2), *i.e.* we have 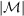 instances that we solve in parallel using our proposed robust estimation procedure of the algorithm given in Section 2.1.2. Among the solutions found, we report the one with the largest number of reciprocal spots for 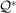 and lower penalization (by using residual threshold annealing, we guarantee that the solutions are below the maximum allowed threshold). The penalization compares the shape of the final basis with the given 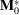 and gives high penalization if the lengths of the vectors or the angles differ by a large margin. Details of this last part of our algorithm can be found in the supporting information.

Note that this approach is capable of handling diffraction patterns corresponding to a few crystals without further iterations, as solutions for each of the crystals can be found independently in different instances.

An important restriction of the presented algorithm is the necessity to have the unit-cell parameters, represented by the matrix 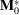, ready beforehand.

## 3. Results

*TORO* was applied to four publicly available protein data sets. In our experiments, we compare *TORO* with *XGandalf* and *MOSFLM*.

The largest and most recent data set is a serial millisecond crystallography data set of lysozyme that was collected on the BioMAX beamline at the MAX IV Laboratory (Leonarski *et al.*, 2023*b* ▸). The crystals were prepared and measured following previously established protocols, with a frame rate of 2 kHz. Additionally, three serial femtosecond crystallography data sets were utilized, namely the serotonin receptor 5-HT_2*B*_ bound to ergotamine (Liu *et al.*, 2013 ▸), β-lactamase (Wiedorn *et al.*, 2018 ▸) and thaumatin (Nass *et al.*, 2021 ▸). These data sets are publicly accessible from the Coherent X-ray Imaging Data Bank (CXIDB) (Maia, 2012 ▸) under entries 21, 83 and 180, respectively. Every data set was analysed under two conditions: with and without the default checks (--no-retry --no-refine --no-check-cell) in the *indexamajig* program, as suggested by Gevorkov *et al.* (2019 ▸). For the CXIDB entries, the data processing protocols as reported in the original publications were adhered to. The processing parameters for lysozyme were fine-tuned to enhance the indexing rate of *XGandalf*.

### 3.1. Indexing rate

Table 1 ▸ presents the indexing rate (in %) of the three indexers (*TORO*, *XGandalf* and *MOSFLM*) for the four proteins with default checks in *indexamajig* enabled (upper panel) and disabled (lower panel). When default checks are enabled *TORO* indexes more patterns than *XGandalf* (0.6%, 8.9% and 5% more) for the three proteins lysozyme, CXIDB ID 83 and CXIDB ID 180, respectively. For CXIDB ID 21, both indexers (*TORO* and *XGandalf*) show the same indexing rate. In all four systems, *MOSFLM* shows a lower indexing rate (∼2–20% less) than both *TORO* and *XGandalf*. For lysozyme, the analysis with default checks on was done with the --multi flag on as well, due the nature of this specific data set (Leonarski *et al.*, 2023*a* ▸). Typically, default sanity measures are enabled in *indexamajig*, such as retry indexing, prediction refinement and cell parameter verification, to ensure enhanced quality of the final result. However, by employing the flags --no-retry, --no-refine and --no-check-cell, this analysis shifts focus to the unadulterated performance of the bare indexing algorithms. When the default checks are disabled (see Table 1 ▸, lower panel) similar behaviour is shown: *TORO* indexes considerably more patterns than *XGandalf*, especially for CXIDB ID 83 (31.5%) and CXIDB ID 180 (48.3%).

In both scenarios – whether the default checks in *indexamajig* are enabled or disabled – *TORO* consistently exhibits comparable or better indexing rates across the data sets.

### 3.2. Merged data quality

**Figure 4:**
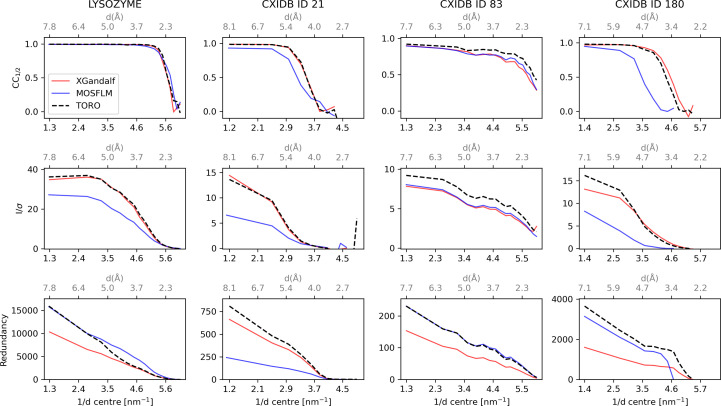
Visualization of various indexing quality metrics for the different systems considered in this study. The rows, from top to bottom, represent the *CC* versus Resolution, *I*/σ versus Resolution and Redundancy versus Resolution subplots, respectively. The columns, from left to right, represent the lysozyme, CXIDB ID 21, CXIDB ID 83 and CXIDB ID 180 systems, respectively. The red, blue and dashed black lines in each plot represent the measured indexing quality of *XGandalf*, *MOSFLM* and *TORO Indexer*, respectively. The plots are obtained from stream files produced with *indexamajig* using --no-retry --no-refine --no-check-cell flags to emphasize the difference in quality between the indexing algorithms.

**Figure 5:**
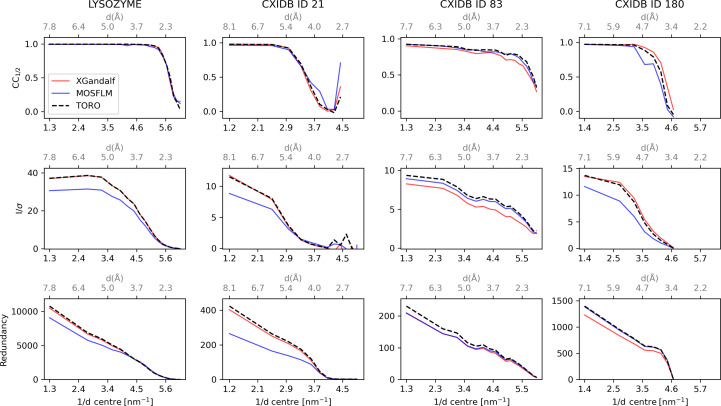
Various quality metrics for the different systems considered in this study, with default sanity checks in *indexamajig* enabled. The rows, from top to bottom, represent the *CC* versus Resolution, *I*/σ versus Resolution and Redundancy versus Resolution subplots, respectively. The columns, from left to right, represent the lysozyme, CXIDB ID 21, CXIDB ID 83 and CXIDB ID 180 systems, respectively. The red, blue and dashed black lines in each plot represent the measured quality of *XGandalf*, *MOSFLM* and *TORO Indexer*, respectively. This analysis presents a comprehensive overview of the data quality metrics under the standard operating conditions of *indexamajig*.

After the diffraction patterns have successfully been indexed and integrated, they are merged to obtain a complete data set, which can be further used for downstream map generation and structure refinement. The patterns were merged and analysed with the programs *partialator* and *compare_hkl* (see Section S1 of the supporting information for more details) of the *CrystFEL* suite (Gevorkov *et al.*, 2019 ▸). Higher indexing rates should lead to improved merging statistics. Therefore, the data quality indicators *CC*_1/2_ (Karplus & Diederichs, 2012 ▸) and *I*/σ (Maes *et al.*, 2008 ▸), in combination with the redundancy of the reflections, were used to assess the indexing quality of *TORO* in comparison with *XGandalf* and *MOSFLM*. As proposed by Gevorkov *et al.* (2019 ▸), the indexing step in *indexamajig* was done with the default checks disabled (flags --no-retry --no-refine --no-check-cell) in order to ensure a fair comparison between the indexers. Results for the four proteins are reported in Fig. 4 ▸. Results with the default checks enabled can be found in Fig. 5 ▸. On average, *TORO* performs similarly to *XGandalf* and outperforms *MOSFLM* for all four cases, as expected from earlier studies with *XGandalf* and *MOSFLM* (Gevorkov *et al.*, 2019 ▸):

For lysozyme, *TORO*’s indexing quality closely mirrors *XGandalf*’s when considering parameters such as redundancy, *I*/σ and *CC*_1/2_. *TORO* indexes ∼10% more patterns than *XGandalf* (Table 1 ▸), which is reflected by the increased redundancy and a slightly improved *I*/σ for the low-resolution range. *TORO* also shows a significantly higher *I*/σ than *MOSFLM*, whereas *CC*_1/2_ is similar for all three indexers.

For CXIDB ID 21, *TORO* shows a similar indexing quality to *XGandalf* in terms of all three statistical metrics, while it shows higher quality for all three metrics than *MOSFLM*.

For CXIDB ID 83, both data quality indicators *CC*_1/2_ and *I*/σ show higher values in all resolution shells compared with *XGandalf*, in accordance with the higher redundancy. *TORO* consistently outperforms *MOSFLM* on all metrics.

For CXIDB ID 180, *TORO Indexer* also shows a similar behaviour to *XGandalf* in terms of *CC*_1/2_, although *XGandalf* performs slightly better in the high-resolution range. *TORO* shows a significantly higher (twice as high) redundancy, which in this case does not translate into a uniform improvement in terms of *I*/σ but only for the low-resolution range (up to ∼5 Å).

In order to validate the previously mentioned metrics, the merged data sets of all proteins for the two indexers *TORO* and *XGandalf* were refined minimally with *PHENIX* (Liebschner *et al.*, 2019 ▸) against the originally published PDB models, showing very similar *R*_work_ and *R*_free_ (see Section S2 in the supporting information).

**Figure 6:**
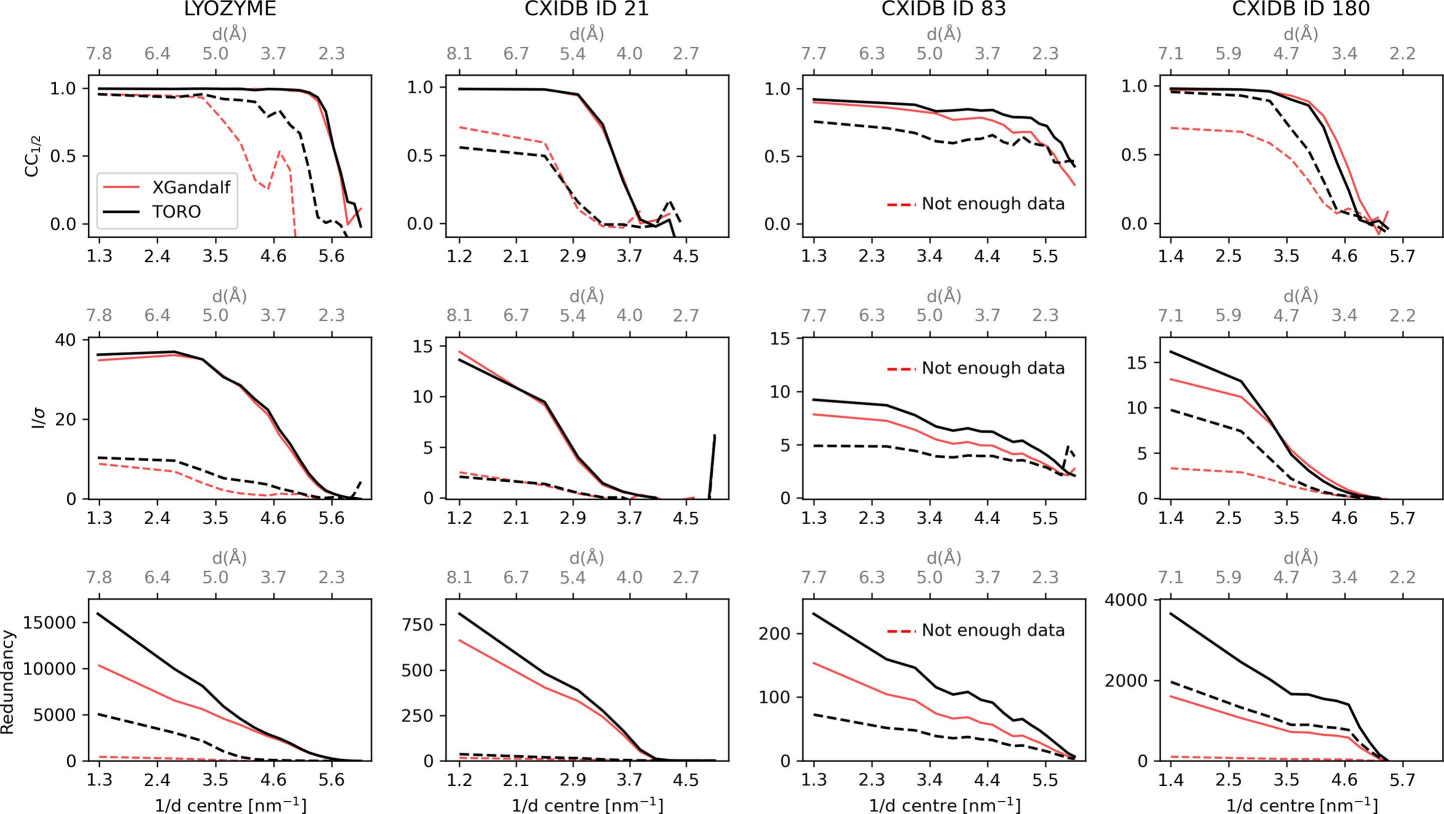
Comparison between *TORO* and *XGandalf* for frames exclusively indexed by one method (and discarded by the other) in different proteins (dashed line) against the full analysis reported in Fig. 4 (solid line). Rows showcase *CC* versus Resolution, *I*/σ versus Resolution and Redundancy versus Resolution metrics, while columns differentiate between lysozyme, CXIDB ID 21, CXIDB ID 83 and CXIDB ID 180. Solid lines represent the measured quality of the indexing algorithm on the full data set, while dashed lines indicate the quality metrics computed only on the subset of frames exclusively indexed by each indexer: black for *TORO* and red for *XGandalf*. Data were generated using *indexamajig* with --no-retry --no-refine --no-check-cell.

The indexing quality is further compared with that of *XGandalf* in Fig. 6 ▸ and Table 2 ▸, showing the subset of frames exclusively indexed by either *TORO* or *XGandalf*. The three metrics demonstrate that the patterns uniquely indexed by *TORO* add meaningful data to the full data set. Notably, *TORO*-indexed frames deliver significant signals for all proteins. *XGandalf* identifies some additional frames for lysozyme, CXIDB ID 21 and CXIDB ID 180. However, its indexing quality there is inferior to that of *TORO*, except for CXIDB ID 21 where they are comparable in terms of *I*/σ and redundancy, while *XGandalf* has a better *CC* at low resolution. Frames exclusively indexed by *XGandalf* for CXIDB ID 83 were omitted, as the number of frames exclusively indexed by *XGandalf* (and not by *TORO*) was insufficient to produce meaningful statistics.

### 3.3. Comparison of estimated profile radii

Another metric obtained from the indexed diffraction patterns is the profile radius of the Bragg spots. This is defined as the maximum distance of a reciprocal-lattice point from the Ewald sphere that can still result in a Bragg reflection (Gevorkov *et al.*, 2019 ▸). This characteristic can be viewed as a property of the crystal and it is influenced by factors such as mosaicity and crystal size. *CrystFEL* estimates this measure from the detected Bragg spots and the reciprocal-lattice points that best predict them. Errors in the indexing solution typically impact the determination of the profile radius (Gevorkov *et al.*, 2019 ▸).

**Figure 7:**
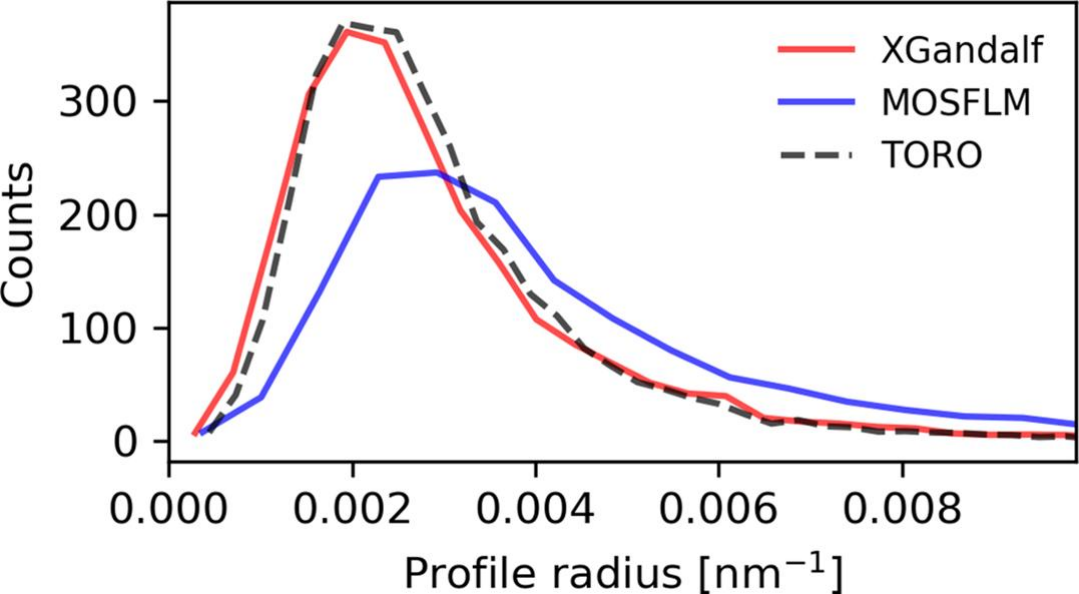
Comparison of the estimated profile radii between *MOSFLM*, *XGandalf* and *TORO Indexer* for CXIDB ID 83, with default checks in *indexamajig* turned off. Patterns indexed by *XGandalf* and *TORO* have smaller estimated radii than those of *MOSFLM*, indicating more precise indexing solutions, in agreement with the results reported in Fig. 10 of Gevorkov *et al.* (2019 ▸)

As demonstrated in Fig. 7 ▸, *TORO*’s indexing quality for CXIDB ID 83 matches that of *XGandalf* and surpasses that of *MOSFLM* [as shown previously in Fig. 10 of Gevorkov *et al.* (2019 ▸)]. The same trend is observed for the remaining three proteins, indicating that the indexing solutions of *TORO* and *XGandalf* are comparable. The estimated mean and standard deviation of the radius profile for CXIDB ID 83 are summarized in Table 3 ▸. As can be inferred, a smaller profile radius indicates better indexing quality, with *TORO* aligning closely with *XGandalf* and outperforming *MOSFLM*.

### 3.4. Implementation details

The stand-alone implementation of *TORO* is coded in Python and consists of less than 500 lines of code. We rely on the *PyTorch* framework, which is designed to exploit raw computing power by enabling parallelism, either on GPUs or on CPU multi-cores. A cornerstone of *PyTorch*’s coding principles is the use of large batches, the elements of which are processed in parallel. For *TORO* this is not straightforward, as different frames have different numbers of strong reflections. While not a problem for sequential CPU processing, this mismatch in the size of the data needs to be addressed to benefit from using large batches. We opted to set a fixed number of spots for each batch, which means that frames with a lower number of spots need to be padded with zeros and frames with more spots need to be pruned. The latter can be done by sorting the spots by resolution and keeping the ones with lower resolution. The maximum number of spots within the frames in a batch has a tangible impact on speed, which is shown in Fig. 9, where we can see a near-linear increase in running time with respect to the size of the batch. It is, however, a common practice among indexers to choose only strong reflections with low resolution. Our speed results revolve around frames with 80 strong reflections, which is above the average in many SX experiments.

Our indexer is encapsulated by a *PyTorch*nn.Module and serialized into a .pt file using *LibTorch* (Paszke *et al.*, 2019 ▸), which can later be loaded into C++ code bases. This is how we are able to develop a *CrystFEL* plug-in that loads this model and integrates it into their pipeline. While this might not be the best way to maximize performance, it allowed us to benchmark the quality of our indexer using the integrated tools within *CrystFEL*. See the Appendix *A*[App appa] for more details of *TORO*’s implementations.

## 4. Computational performance analysis

In this section, we benchmark the computational performance of *TORO*. First within *CrystFEL*, with the --profile option enabled, we conducted a thorough performance comparison between *TORO* and *XGandalf* (Gevorkov *et al.*, 2019 ▸). This particular option provides granular insights into execution times at every step of the analysis. For this comparison, both indexing algorithms were evaluated on a subset of the lysozyme data set. Specifically, our benchmarking data set consists of 953 frames, containing only frames indexable by both *TORO* and *XGandalf* and each having exactly 80 strong reflections. These reflections were identified using the *peakfinder8* tool, with the detailed parameters outlined in Section S4 of the supporting information. In an SX experiment, many frames might be empty or have weak reflections, while some might contain multiple crystals, so performance is heavily dependent on the data set used. We believe that our benchmarking data set describes a realistic but at the same time challenging setting meaningful for this benchmarking task. A stream file corresponding to our benchmarking data set is included in the released code.

*TORO* was incorporated into *CrystFEL* using *LibTorch* (Paszke *et al.*, 2019 ▸) and serialized .pt models. Both tools were tested under the same computational conditions to ensure a fair comparison. Specifically, they were both run on a single core (as stipulated by the -j1 flag) of an Intel Xeon Gold 6230R CPU running at 2.1 GHz. The flags --no-revalidate --no-retry --no-refine --no-check-peaks were used in all the *CrystFEL* benchmarks.

**Figure 8:**
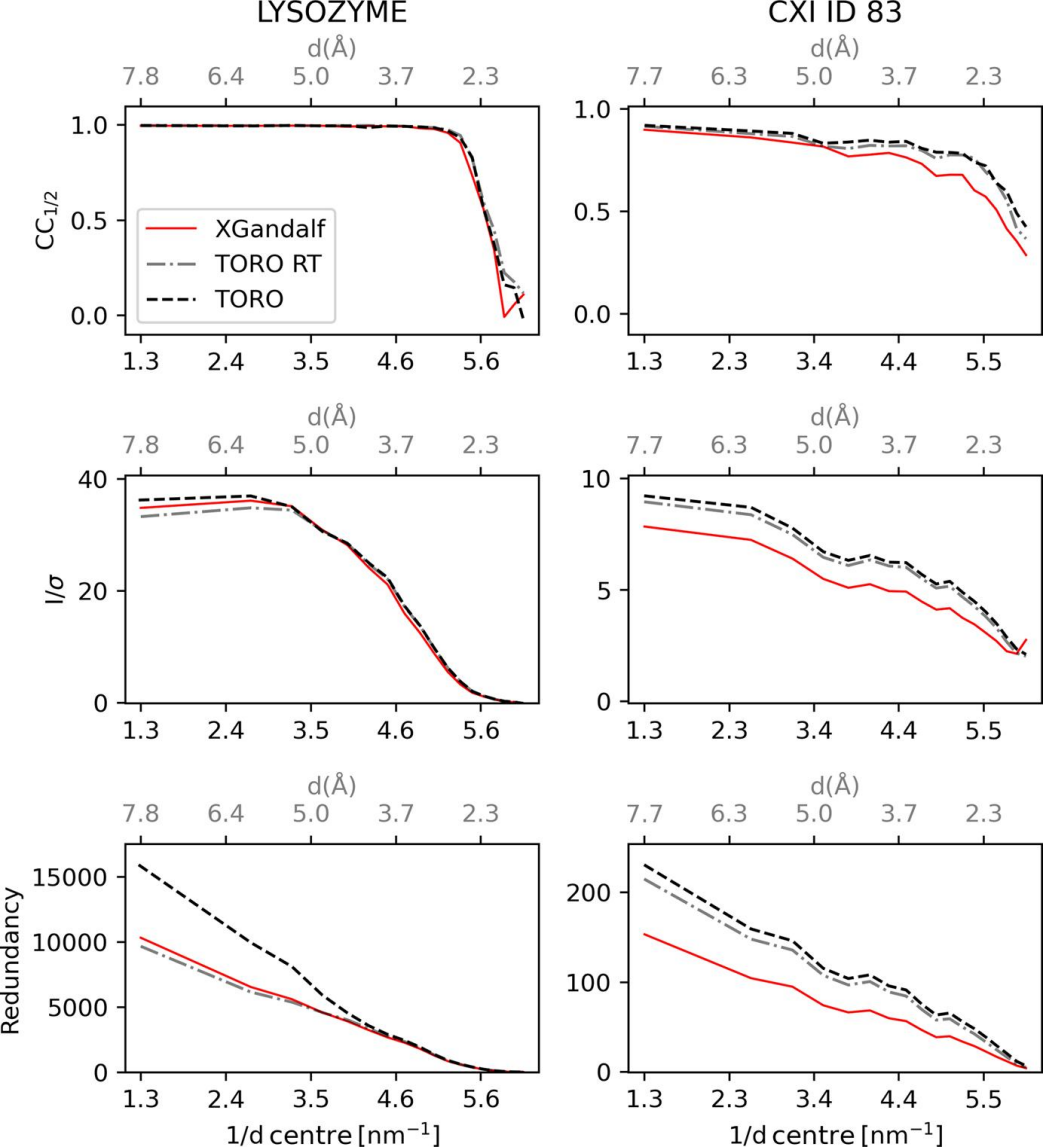
Indexing quality profiling of *TORO* (black dashed line) versus *XGandalf* (red solid line) across the lysozyme and CXIDB ID 83 data sets, exploring varying *TORO* hyperparameters for optimal speed. Metrics presented include *CC* versus Resolution, *I*/σ versus Resolution and Redundancy versus Resolution. Distinct columns represent data from the lysozyme and CXIDB ID 83 data sets, respectively. For context, findings from Fig. 4 for both *TORO* and *XGandalf* are juxtaposed against *TORO* RT (dashed–dotted grey line) – a swifter yet slightly less precise variant of *TORO*, configured with lattice_size=10000, angle_resolution=100 and num_top_solutions=25. *TORO* RT boasts a processing rate of 3006.76 images s^−1^ on an A100 GPU in standalone mode, making it a suitable candidate for real-time feedback indexing. Quality-wise, *TORO* RT remains robust, matching (lysozyme) or outperforming (CXIDB ID 83) *XGandalf*. Data sets were processed using *indexamajig* with --no-retry --no-refine --no-check-cell.

The key performance indicator evaluated was the computational time for indexing (time for the indexing routine, as reported by the profiling option). On average, *XGandalf* took 404 ms (standard deviation 8.2 ms) for indexing, while *TORO* demonstrated inferior speed with 621 ms (standard deviation 49 ms). This performance was obtained using the flag --xgandalf-fast-execution for *XGandalf* and by using the parameters lattice_size=50000, angle_resolution=150 and num_top_solutions=400 for *TORO*. When decreasing the parameters to lattice_size=10000, angle_resolution=100 and num_top_solutions=25 – which we dub *TORO* real time (RT) – the performance of *TORO* improves considerably (without sacrificing quality, as reported in Fig. 8 ▸), reducing the indexing time to 53 ms (standard deviation 66 ms). The average execution times for the indexing part of an *indexamajig* cycle were 1.73, 1.00 and 2.16 images s^−1^ for *XGandalf*, *TORO* and *TORO* RT , respectively. We are confident that, with a more refined implementation targeted at optimizing existing bottlenecks within our prototype *CrystFEL* plugin, the overall performance of the *TORO* plugin will improve significantly in the future.

**Figure 9:**
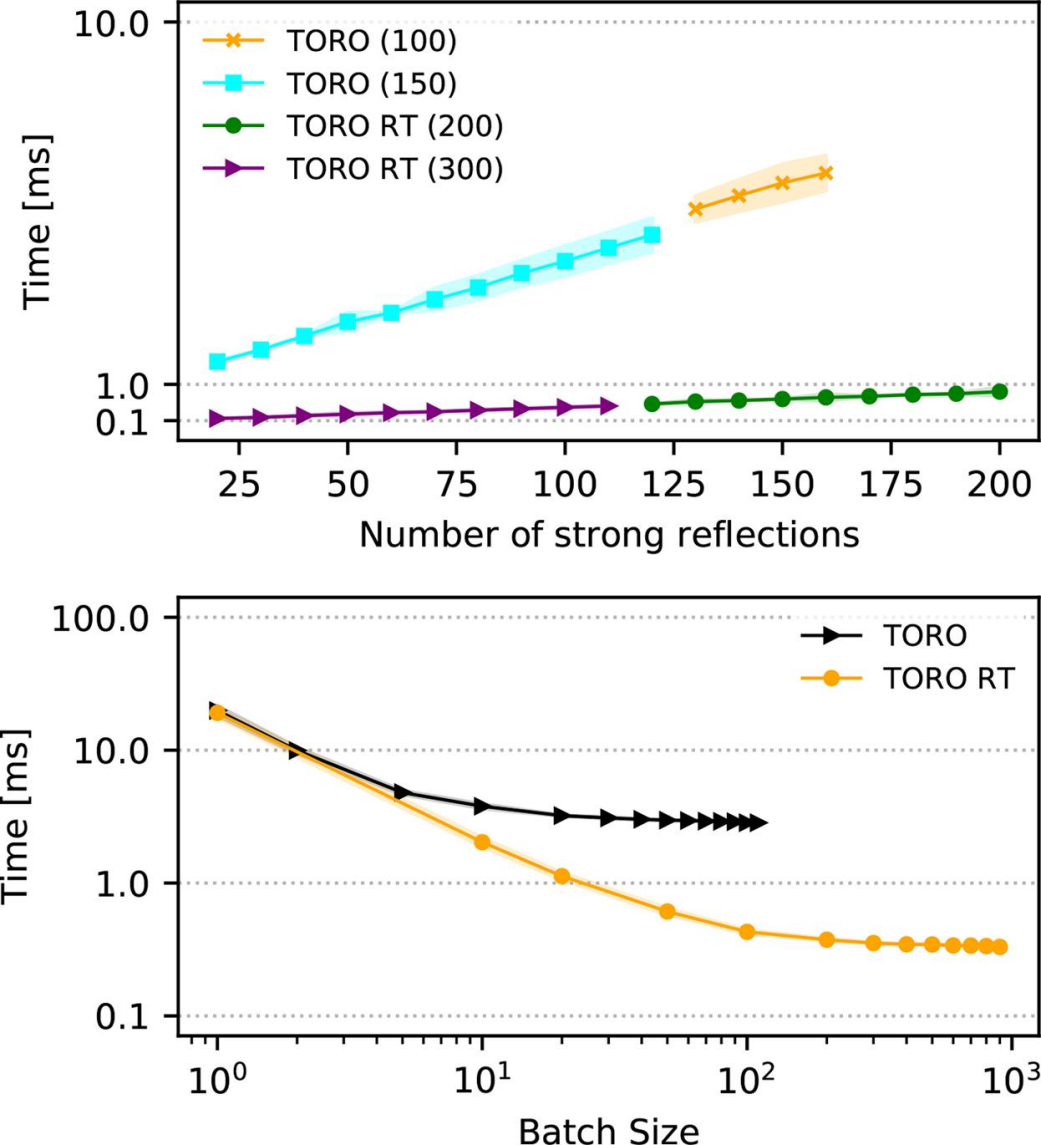
The performance of *TORO Indexer*. (Upper panel) Average execution time (milliseconds) against the number of strong reflections. The plots compare different models, *TORO* and *TORO* RT, and the batch size used (shown in brackets). The shaded regions represent the standard deviation around each curve. (Lower panel) Average execution time (milliseconds) as a function of batch size.

The true potential of *TORO* is unveiled when operated as a standalone application, capitalizing on the combination of Python’s expressiveness and ease of use along with *PyTorch*’s robust computational capabilities, especially when deployed on GPU architectures. Table 4 ▸ reports results for *TORO* and *TORO* RT when running on the high-end NVIDIA A100 GPU. Indexing our benchmarking data set, *TORO* achieves an indexing speed of 301.21 images s^−1^ (standard deviation 33.09 images s^−1^) while *TORO* RT runs in the kilohertz regime, processing 3006.76 images s^−1^ (standard deviation 24.68 images s^−1^). In order to achieve these speeds, *TORO* processes spot patterns within large batches in parallel: up to 900 for the aforementioned result. Batching allows us to use all resources from the GPU. In practice, we recommend setting the batch size to the largest value possible allowed by the GPU memory (Fig. 9 ▸). This implies that the pipeline executing the indexing in real time would need a buffer to store all the frames of one batch before passing the batch to the indexer. In order to measure the speed with such large batch sizes more reliably, we stack copies of the benchmark data set until surpassing 10 000 frames and then trim this new stacked data set until its size becomes a multiple of the batch size. In this way, we can average the performance over several batches and we ensure that each tested batch has the same number of elements.

Fig. 8 ▸ shows a quality profile comparison between *TORO* and *XGandalf*. This comparison emphasizes distinct variations in indexing quality for both the lysozyme and CXIDB ID 83 data sets. Intriguingly, *TORO* RT, which is optimized for speed, either equates to or excels over *XGandalf*, underscoring its efficiency. Fig. 9 ▸ offers further insights with an in-depth analysis of *TORO*’s computational performance. The upper panel elucidates the interplay between average execution time and the number of strong reflections (with fixed batch size). The lower panel shows the significant influence of batch size on performance. A larger batch size allows for more data points to be processed simultaneously, exploiting the GPU’s capability to handle multiple operations in parallel and maximizing its throughput.

## 5. Discussion and conclusions

Modern advances in SX necessitate indexing algorithms that can handle significant data volumes with precision. Traditional methodologies, while robust, often struggle to keep up with the escalating data rates of modern high-performing detectors. To counter this, we have introduced *TORO*, a novel indexing algorithm optimized for modern GPUs, applicable when unit-cell parameters are known. *TORO* exhibits superior speed while maintaining data quality and showing equal or higher indexing rates.

Our analysis of four protein data sets highlights the equal or superior indexing quality of *TORO* compared with *XGandalf*, one of the most prominent indexers used in SX. Overall *TORO* achieved significantly higher redundancy, reflecting the detection of a larger number of patterns. Importantly, this increased pattern detection did not result in a degradation of data quality, as shown by the sustained high *I*/σ from uniquely indexed frames. These findings underscore the robustness and efficacy of *TORO* as an indexing algorithm for SX data.

This level of computational performance opens a few doors for the SX community. On the one hand, indexing is no longer the bottleneck to obtaining real-time feedback during an experiment, which can provide valuable information to beamline users. On the other hand, one could consider indexing in real time and storing only indexable frames, which could help reduce the large amounts of data stored in SX experiments. While this requires further research, our findings suggest that using our indexer to discard non-indexable frames would preserve enough information to obtain similar reconstructions to those obtained using current pipelines based on *XGandalf*.

*TORO*’s implementation in Python and *PyTorch* further enhances its appeal. This implementation facilitates ease of maintenance and updates and scalability across CPUs and GPUs, and capitalizes on the wide array of libraries and tools offered by the modern AI development stack. The streamlined code base of a few hundred lines delivers a state-of-the-art performance, highlighting the remarkable advantages introduced by *PyTorch* in scientific software development. The ease of use of this code base provides developers with the ability to modify and tailor this indexer further to their specific needs.

We have made a preliminary integration of *TORO* into the *CrystFEL* suite to enhance its accessibility within the crystallographic community and allow for a comparison with other indexers. This will not only allow users to make use of *TORO*’s speed and precision but also facilitate access to the comprehensive set of tools provided by *CrystFEL*. Standalone versions of *TORO* are readily portable to other dedicated software environments on beamlines, thereby augmenting its potential utility. The simplicity of the *TORO* code base allows adaptation of the algorithm by scientists for additional indexing problems, like pink-beam or two-colour SX, electron diffraction, or rotational crystallography.

As with all newly introduced methodologies, *TORO* is not without its limitations. While it shows promise in terms of speed and flexibility, rigorous benchmarking under diverse data sets, experimental conditions and integration scenarios is still warranted. Initial implementations within *CrystFEL* have revealed performance bottlenecks that need addressing. An important design choice for *TORO* is the required estimate of the crystal unit cell. Our goal is to provide a highly robust solution for automated indexing and we see this mostly possible for a simple use case of a well defined protein. Our experience is that more complex cases, *e.g.* crystal contamination or unknown or multiple crystal forms, should be understood with offline processing and are not limited by raw throughput. We see existing algorithms (like *MOSFLM* or *XGandalf*) as more suitable for these cases. Furthermore, to achieve a high indexing speed we need to use batching, which might increase the complexity of a real-time pipeline and limits the performance of our *CrystFEL* plugin.

In conclusion, preliminary findings suggest that *TORO* offers competitive indexing quality and superior speed against established algorithms like *XGandalf*. Its modular design aims to streamline updates and modifications, which can be advantageous in the dynamic field of crystallography. However, consistent evaluations and user feedback will be critical in refining *TORO*, ensuring its robustness and confirming its potential value in future SX research.

## 6. Related literature

The following additional literature is cited in the supporting information: González (2010 ▸).

## Supplementary Material

Supporting Information. DOI: 10.1107/S1600576724003182/jo5098sup1.pdf

## Figures and Tables

**Figure 10:**
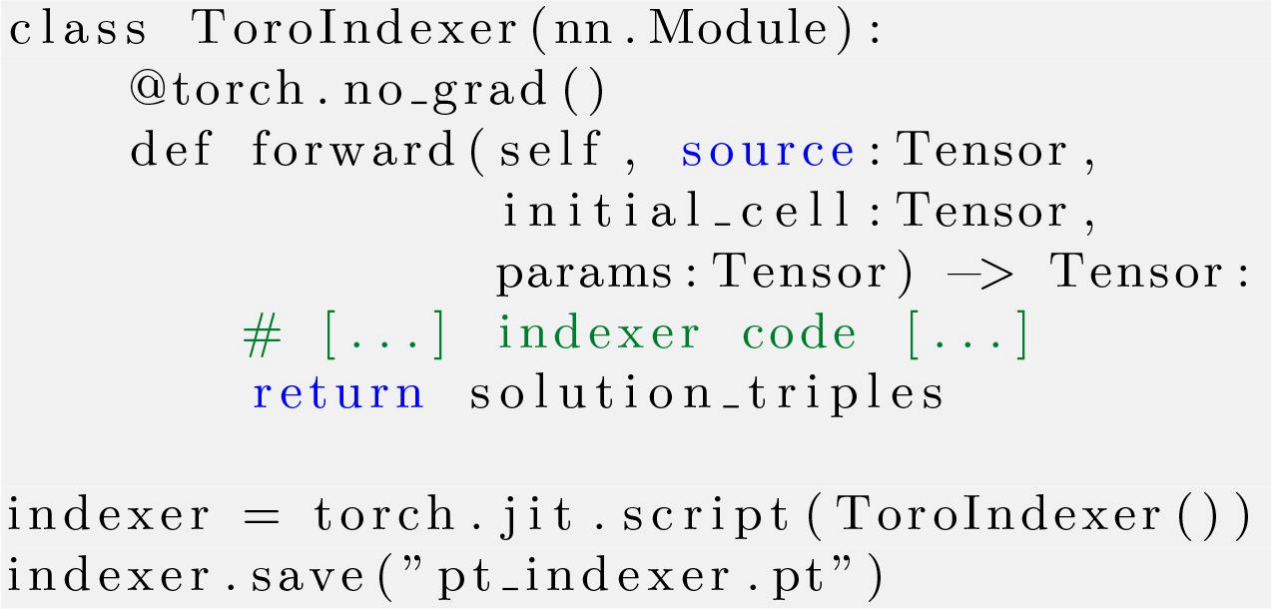
Listing 1. *TORO* follows the general implementation and serialization in *PyTorch*. The torch.jit.script translates the model to *TorchScript*, allowing it to run without Python. The serialized model is saved to disk for later use without the original Python code. Indexer specifics, as detailed in the main text, are omitted, but this *PyTorch* interface can be used for any indexer algorithm, allowing it to be integrated into *CrystFEL* without recompiling the application.

**Figure 11:**
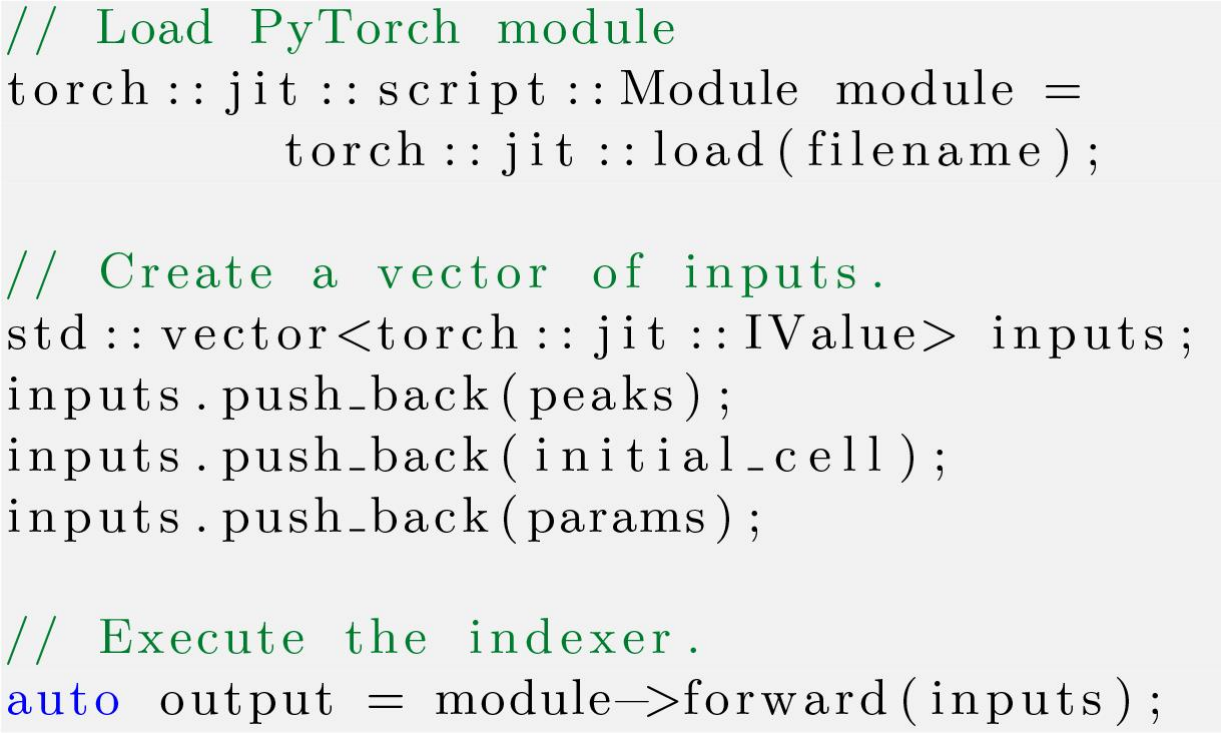
Listing 2, an example integration of a serialized *PyTorch* model into the *CrystFEL* suite. Using *LibTorch*, the serialized model is loaded, the necessary inputs are prepared and the indexer is then executed. This interface highlights the potential for using serialized *PyTorch* models within a C++ application, allowing integration without recompilation.

**Figure 12:**
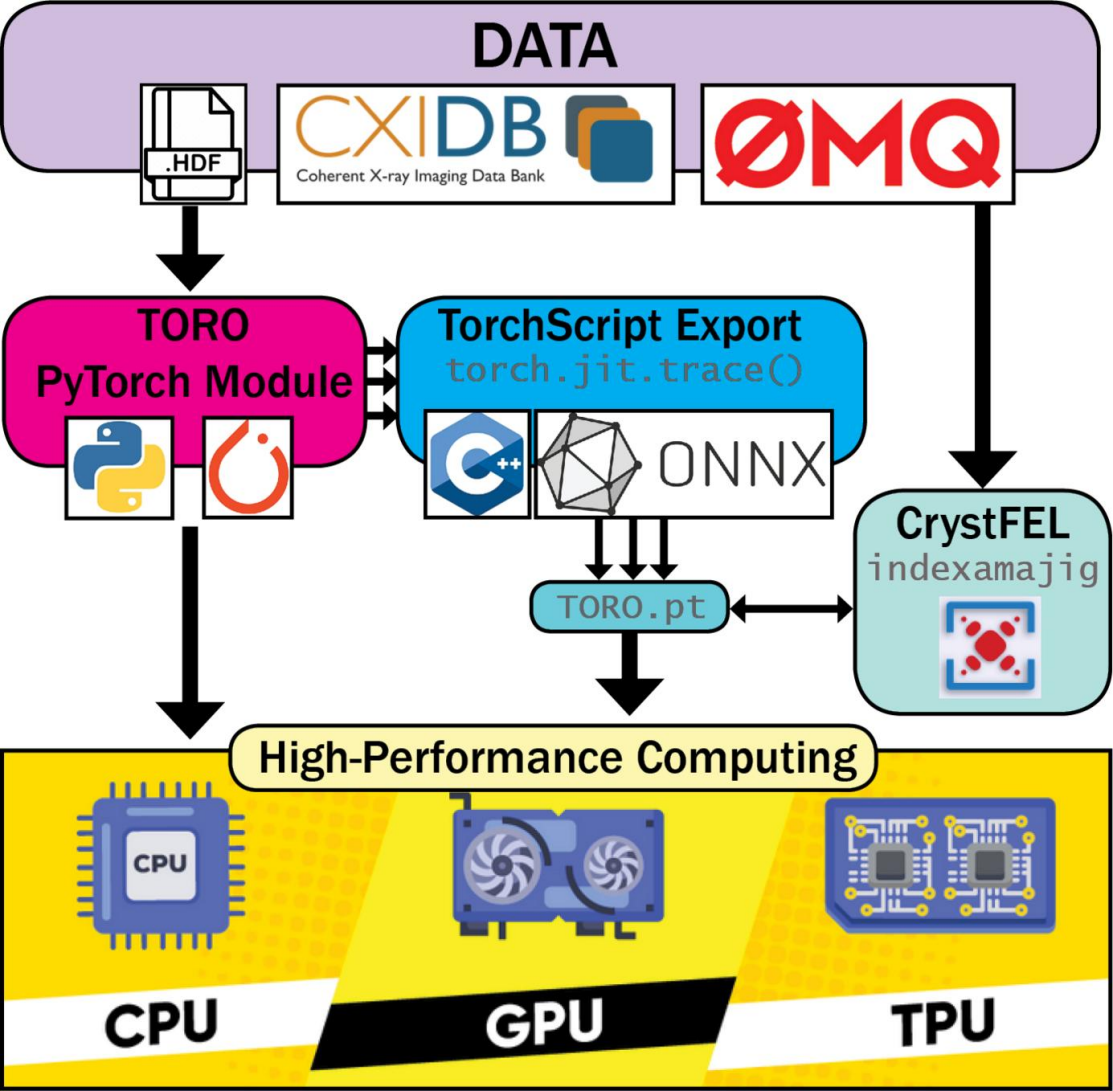
A schematic representation of the full *TORO* pipeline. Users can use *TORO* as a standalone Python module or use *TorchScript* to run it in a non-Python environment, *e.g.CrystFEL*. *TORO* comes with a flexible implementation and its performance and functionality remain consistent regardless of the data transport method used, be it through the file system, 0MQ or any alternative approach.

**Table:** Numbers in bold highlight the best performing algorithm for each system.Default checks ON.

Protein (processed frames)	*TORO*	*XGandalf*	*MOSFLM*
Lysozyme (500 000 frames)	**53.3**	52.7	32.0
CXIDB ID 83 (16 532 frames)	**93.8**	84.9	79.2
CXIDB ID 21 (152 531 frames)	**10.2**	**10.2**	5.1
CXIDB ID 180 (59 928 frames)	**53.2**	48.2	46.2

**Table:** Default checks OFF.

Protein (processed frames)	*TORO*	*XGandalf*	*MOSFLM*
Lysozyme (500 000 frames)	**60.0**	50.5	29.8
CXIDB ID 83 (16 532 frames)	**90.5**	59.0	69.6
CXIDB ID 21 (152 531 frames)	**18.1**	16.7	5.7
CXIDB ID 180 (59 928 frames)	**99.5**	51.2	59.3

**Table 2:** Frames uniquely indexed by one indexer and discarded by the other

Protein	*TORO*	*XGandalf*
Lysozyme	64845	17591
CXIDB ID 21	9101	5233
CXIDB ID 83	5279	72
CXIDB ID 180	29406	1883

**Table 3:** Mean and standard deviation of the estimated profile radii of different indexers for CXIDB ID 83

Indexer	Mean ± s.d. (nm^−1^)
*MOSFL*	0.0054 ± 0.0044
*XGandalf*	0.0032 ± 0.0019
*TORO*	0.0031 ± 0.0016

**Table 4:** Performance of *TORO* and *XGandalf* on various hardware accelerators on lysozyme over 953 indexable frames containing 80 strong reflections identified using *peakfinder8* The *CrystFEL* configuration indicates that the performance test was done using *indexamajig* with flags --no-revalidate --no-retry --no-refine --no-check-peaks --no-multi and the --profile flag, while Python refers to standalone Python code. Time is reported in milliseconds.

Hardware accelerator	Type	Configuration	Time (ms)	Speed-up	Batch size
Intel Xeon Gold 6230R	CPU	*CrystFEL* (*XGandalf*)	404	1.0×	1
Intel Xeon Gold 6230R	CPU	*CrystFEL* (*TORO*)	621	0.7×	1
Intel Xeon Gold 6230R	CPU	*CrystFEL* (*TORO* RT)	53	7.6×	1
NVIDIA A100	GPU	Python (*TORO*)	3.3	122.4×	100
NVIDIA A100	GPU	Python (*TORO* RT)	0.33	1214×	900

## Appendix A Data and code

### A1. Program and data availability

The standalone optimized version of *TORO Indexer*, tailored for diverse computational environments, is available at https://renkulab.io/projects/lfbarba/toro-indexer-for-serial-crystallography.

*TORO*’s *CrystFEL* plugin is available at https://github.com/henrique/CrystFEL/tree/torch_indexer.

The CXIDB ID 83, CXIDB ID 21 and CXIDB ID 180 data sets are available under the corresponding IDs at https://cxidb.org/. The lysozyme data set is available at https://doi.org/10.2210/pdb8p1a/pdb.

### A2. Software implementation

*TORO Indexer* is built on the *PyTorch* framework (Paszke *et al.*, 2019 ▸). *PyTorch* allows the *TORO Indexer* code base to be both concise, with fewer than 500 lines, and efficient. It achieves indexing quality comparable to that of *XGandalf* while ensuring portability across all supported accelerators. Additionally, through *TorchScript*, *PyTorch* facilitates seamless integration into C++ and Java software environments. Listing 1 in Fig. 10 ▸ shows a simple example implementation and serialization of *TORO* in Python. We use the torch.jit.script function to map the *PyTorch* model to *TorchScript*, an intermediate representation that eliminates the Python runtime dependency. The *TorchScript* model is then serialized to disk, enabling non-Python-dependent deployment.

To demonstrate the integration process further, Listing 2 in Fig. 11 ▸ offers an insight into how a serialized *PyTorch* model can be loaded and utilized within the *CrystFEL* suite using the *LibTorch* API. These few lines of code are enough to integrate *TORO* into *CrystFEL* without the need for recompiling the application. While this endeavour exemplifies *TORO*’s adaptability potential, it is worth noting that *TORO*’s integration within *CrystFEL* is still in its early stages and would benefit from continuous feedback and refinements from the *CrystFEL* community. An initial implementation of *TORO*’s *CrystFEL* plugin is available at https://github.com/henrique/CrystFEL/tree/torch_indexer.

Fig. 12 ▸ illustrates an example of the end-to-end processing pipeline using *TORO*. It highlights how *TORO* exploits high-performance computing for real-time indexing with its Python implementation and for offline processing using either the Python or *CrystFEL* versions. The depicted pipeline is modular and scalable, ensuring ease of adaptation and integration into high-performance computing infrastructures or edge computing setups across diverse beamlines.
