# Supplementary material for: TORO Indexer: a PyTorch-based indexing algorithm for kilohertz serial crystallography
Source: J Appl Crystallogr. 2024 Jun 18;57(Pt 4):931–44. doi: 10.1107/S1600576724003182 (PMC11299607; doi:10.1107/S1600576724003182)
Supplement: Supplementary file 1 [file j-57-00931-sup1.pdf]

JOURNAL OF APPLIED CRYSTALLOGRAPHY

Volume 57 (2024)

Supporting information for article:

TORO Indexer: a *PyTorch*-based indexing algorithm for kilohertz serial crystallography

Piero Gasparotto, Luis Barba, Hans-Christian Stadler, Greta Assmann, Henrique Mendonça, Alun W. Ashton, Markus Janousch, Filip Leonarski and Benjamín Béjar

S1. CrystFEL commands

This section provides a detailed rundown of the commands used to process data for each system in our study. For each system, we lay out the necessary prerequisites, the indexing process, and the steps for merging and scaling. All the datasets were processed using with CrystFel 0.10.2. The indexing is with `indexamajig`, merging and scaling with `partialator` and the statistics are computed using `compare_hkl`. Here below we show the example for XGandalf, disabling all the default sanity checks in `indexamajig`. To switch the sanity checks on, the flags `--no-retry`, `--no-refine`, and `--no-check-cell` should be removed.

S1.1. Lysozime

Indexing:

```
cellfile=lyso.cell
indexamajig --peaks=peakfinder8 --indexing=xgandalf --xgandalf-fast-
    execution --threshold=10 --int-radius=2,3,5 -p ./lyso.cell --min-snr
    =3.0 --min-peaks=6 --min-pix-count=1 -i list -o Lyso_XGandalf.stream
    -g ./jf4m.geom -j${NPROC} --min-res=75 --no-retry --no-refine --no-
    check-cell >& log.lyso_xgandalf
```

Only for Lysozime, the analysis with default checks on was done also with the `--multi` flag on.

Merging and Scaling:

```
resolution=1.60
sys_sym=4/mmm
resbins=18
cellfile=lyso.cell
partialator --push-res=1 -i ${input_stream} -o ${output_hkl} -y $sys_sym
    --iterations=1 --model=unity --max-adu=7000
```

S1.2. ID 21

Indexing:

```
indexamajig -i lst -o 5HT2B_XGANDALF.stream -g 5HT2B-Liu-2013.geom -j${
    NPROC} --peaks=zaef --threshold=450 --min-gradient=500 --min-snr=4 --
    indexing=xgandalf --xgandalf-fast-execution -p 5HT2B.cell --tolerance
    =4,4,4,1.4 --int-radius=3,7,8 --no-retry --no-refine --no-check-cell
```

Merging and Scaling:

```
resolution=2.00
sys_sym=mmm
resbins=12
partialator --push-res=1 -i ${input_stream} -o ${output_hkl} -y $sys_sym
    --iterations=1 --model=unity --max-adu=7000
```

S1.3. ID 83

Indexing:

```
indexamajig -i lst -o ID_83.stream -g agipd_mar18_v10.geom -j${NPROC} --
    peaks=cxi --no-revalidate --indexing=xgandalf --xgandalf-fast-
    execution -p protein.cell  --int-radius=2,3,4 --push-res=0.2 --no-
    retry --no-refine --no-check-cell
```

Indexing ambiguities are resolved with the amibigator program:

```
ambigator ID_83.stream -o ID_83.ambi.stream --lowres=3.0 -y -3m1_H -w 6/
```

Merging and Scaling:

```
resolution=1.65
sys_sym=-3m1_H
resbins=18
partialator --push-res=1 -i ${input_stream} -o ${output_hkl} -y $sys_sym
   --iterations=1 --model=unity --max-adu=7000 --no-scale
```

S1.4. ID 180

Indexing:

```
indexamajig --indexing=xgandalf --xgandalf-fast-execution --peaks=
    peakfinder8 --min-snr=4.5 --min-pix=2 --max-pix=60 --threshold=20 --
    int-radius=2,3,5 --min-pix-count=1 -p ./xgdf.cell -i list -o
    ID_180_XGANDALF.stream -g ./JF16M-Alvra-pink.geom --no-revalidate --
    no-retry --no-refine --no-check-peaks --max-res=2000 -j${NPROC}
```

Merging and Scaling:

```
resolution=1.60
sys_sym=4/mmm
resbins=18
partialator --push-res=1 -i ${input_stream} -o ${output_hkl} -y $sys_sym
   --iterations=1 --model=unity --max-adu=7000
```

S2. Refinements

Command:

```
phenix.refine file.mtz file.pdb input.xray_data.labels="FP,SIGFP" input.
    xray_data.r_free_flags.label="FreeRflag" ordered_solvent=false
    strategy=individual_sites+individual_sites_real_space+individual_adp+
    occupancies optimize_xyz_weight=true optimize_adp_weight=true output.
    prefix=refine
```

Refinement results for the different proteins discussed in the main text, for both XGandalf and TORO:

**Protein**	**PDB ID**	**XGandalf**	**TORO**
Lysozyme	4XJD	R_{work} = 0.2349, R_{free} = 0.2720	R_{work} = 0.2313, R_{free}= 0.2683
CXIDB ID 21	4NC3	R_{work} = 0.2520, R_{free} = 0.2968	R_{work} = 0.2546, R_{free} = 0.2907
CXIDB ID 83	6GTH	R_{work} = 0.2483, R_{free}= 0.3019	R_{work} = 0.2538, R_{free} = 0.2965
CXIDB ID 180	7O5J	R_{work}= 0.1795, R_{free}= 0.1961	R_{work} = 0.1749, R_{free} =0.1890

S3. Performance Profiling in `indexamajig`

Figure S1 illustrates three subpanels reporting the time series profiling of both TORO and TORO RT in juxtaposition to Xgandalf.

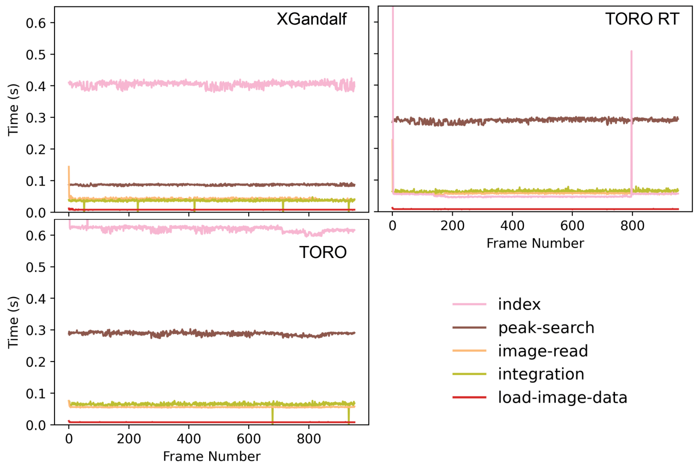

Fig. S1. The three subpanels delineate the distinct profiling time series for TORO and TORO RT in relation to Xgandalf.

Utilizing the `--profile` tag outputs the time taken for each process in stdout. The displayed graphs depict the time per `indexamajig` operation over a series of 953 frames extracted from the Lysozyme dataset. These frames were curated by exclusively selecting those that were indexable by both TORO RT and Xgandalf and contained 80 strong reflections. The pivotal data points in the graphs are:

- **H5Dread:** Loading the HDF5 file.
- **load-masks:** Incorporating the `mask.h5` file.
- **pf8:** The peakfinder 8 procedure which encapsulates the processes pf8-mask, pf8-rstats, and pf8-search.
- **indexing:** The indexing method.

The integration of TORO into CrystFEL was achieved using LibTorch (Paszke *et al.*, 2019) alongside serialized `.pt` models. To maintain fairness in the evaluation,

both tools were subjected to the same computational conditions. Specifically, they were operated on a singular core, as indicated by the `-j1` flag, of an Intel(R) Xeon(R) Gold 6230R CPU running at 2.1 GHz. All CrystFEL benchmarks utilized the flags `--no-revalidate --no-retry --no-refine --no-check-peaks`.

The difference between TORO and TORO RT consists in the parameters used:

- **TORO**:
  - lattice_size = 50000
  - angle_resolution = 150
  - num_top_solutions = 400
- **TORO RT**:
  - lattice_size = 10000
  - angle_resolution = 100
  - num_top_solutions = 25

On average, TORO RT is 7.6 times faster in its indexing routine than Xgandalf, as visualized in Figure S1. However, the overall cycle time per frame in `indexamajig` doesn't display a substantial increase in speed. TORO's average speed is slower by a factor of 0.7 when compared to Xgandalf.

Interestingly, the time consumed by peakfinder8 significantly escalates when `indexamajig` is run with TORO. This spike is possibly attributed to a non-optimized implementation of our libtorch interface, particularly during the transmission of strong reflections as inputs to TORO.

The average execution times for the indexing part of an `indexamajig` cycle were 1.73, 1.00 and 2.16 images/s for XGandalf, TORO and TORO RT respectively.

Below are the exact commands utilized in the performance testing:

TORO:

```
indexamajig --peaks=peakfinder8 --threshold=10 --int-radius=2,3,5 -p ../
    lyso.cell --min-snr=3.0 --min-peaks=6 --min-pix-count=1 -i ../lst -o
    Lyso_TORO_RT_noall_1_cpu.stream -g ../jf4m.geom -j1 --min-res=75 --no
```

```
-retry --no-refine --no-check-cell --indexing=torchidx --torchidx-
    filename=/das/work/p19/p19607/FromGreta/REDML_indx_data/models/
    traced_model_real_time.pt --torchidx-params=12,100,25 --torchidx-
    num-threads=1 --profile >& log.lyso_noall_TORO_RT_1_cpu
```

XGandalf:

```
indexamajig --peaks=peakfinder8 --indexing=xgandalf --xgandalf-fast-
    execution --threshold=10 --int-radius=2,3,5 -p ./lyso.cell --min-snr
    =3.0 --min-peaks=6 --min-pix-count=1 -i lst -o
    Lyso_XGandalf_noall_1_cpu.stream -g ./jf4m.geom -j1 --min-res=75 --no
    -retry --no-refine --no-check-cell --profile >& log.
    lyso_noall_xgandalf_1_cpu
```

S4. Sampling can replace the oracle

In this section, we present the details our algorithm uses to replace the oracle. Recall that we presented an algorithm to solve the indexing problem that relies on an oracle which provides the true Miller indices for the reciprocal spots in Q. While this is seemingly a strong assumption, in this section we show that with enough computing power, a sampling strategy can play the role of this oracle. Recall that we assume that the structure of the ideal crystal lattice basis vectors is given, i.e., we know the ideal norms of a, b, c and the ideal angles between them (the actual solution might differ slightly from these ideal conditions). Thus, we can assume that we are given a matrix $\boldsymbol{M}_0^\star := (\mathbf{a}_0^\star, \mathbf{b}_0^\star, \mathbf{c}_0^\star)^T$, being the given ideal crystal lattice basis with an arbitrary orientation.

Notice that if we are given an arbitrary rotation of $\boldsymbol{M}_0^\star$, say $\boldsymbol{M}'$, it *induces* a set of possible Miller indices for the reciprocal spots of $\mathcal{Q}$ by considering the set $\{\text{ROUND}(\boldsymbol{M}\boldsymbol{q}) : \boldsymbol{q} \in \mathcal{Q}\} \subset \mathbb{Z}^3$. Recall that for each $\boldsymbol{q} \in \mathcal{Q}^*$, $\boldsymbol{v}_q = (h_q, k_q, l_q)$ denotes the true set of Miller indices of $\boldsymbol{q}$. The goal of our sampling strategy is to construct candidates basis being rotations of $\boldsymbol{M}_0^\star$ such that at least for one of them, the induced Miller indices of this basis mostly coincides with the true Miller indices of $\mathcal{Q}^*$. That is, we want to sample a basis $\hat{\boldsymbol{M}}$ such that $\text{ROUND}(\hat{\boldsymbol{M}}\boldsymbol{q}) = \boldsymbol{v}_q$ for most reciprocal

spots $\boldsymbol{q} \in \mathcal{Q}^*$. If that were the case, then by running our robust optimization on each candidate basis, the optimization will succeed in solving the indexing problem for $\hat{\boldsymbol{M}}$.

To construct the basis samples, our algorithm starts by sampling single vectors from the surface of the sphere to be candidates for the crystal lattice basis vector $\boldsymbol{a}^*$ (the process is repeated in parallel for $\boldsymbol{b}^*$ and $\boldsymbol{c}^*$, but we describe it only for $\boldsymbol{a}^*$ for simplicity). We then choose the most promising candidates for $\boldsymbol{a}^*$ and use them to construct several candidate bases by attaching $\boldsymbol{b}$ and $\boldsymbol{c}$ using the provided structure of the crystal lattice basis $\boldsymbol{M}_0^\star$. We formalize this as follows.

Let $\mathcal{A} \subset \mathcal{S}^2$ be a "well-spread" sample of k points on the boundary of the sphere of radius $\|\mathbf{a}^\star\|$ using the Fibonacci lattice spiral (González, 2010). The set $\mathcal{A}$ contains candidates for the crystal lattice basis vector $\boldsymbol{a}^*$. We want to define a score for the vectors of $\mathcal{A}$ that ranks higher those vectors where the Laue condition is "closer" to being satisfied. To this end, for a fixed hyperparameter δ, let

$$\phi_\delta(\boldsymbol{x}) = \begin{cases} 1 & \text{if } \|\boldsymbol{x} - \textsc{round}(\boldsymbol{x})\| \leq \delta \\ 0 & \text{otherwise} \end{cases}$$

The score function for each $\boldsymbol{a} \in \mathcal{A}$ is given by

$$\textsc{score}(\boldsymbol{a}) := \sum_{\boldsymbol{q} \in \mathcal{Q}} \phi_\delta\left(\boldsymbol{a} \cdot \boldsymbol{q}\right),$$

which counts how many reciprocal spots in $\mathcal{Q}$ have their dot product with $\boldsymbol{a}$ being at most δ away from its closest integer, i.e., they are "δ-close" to satisfying the Laue condition. We rank the candidates vectors in $\mathcal{A}$ according to their value $\textsc{score}(\boldsymbol{a})$ and keep only the top t of them.

We now say that each vector $\boldsymbol{a} \in \mathcal{A}$ proposes a Miller index $h_{\boldsymbol{a},\boldsymbol{q}} := \textsc{round}(\boldsymbol{a}^\top \boldsymbol{q})$ for each reciprocal spot $\boldsymbol{q} \in Q$. Our hope is that with enough samples, some of the vectors in $\mathcal{A}$ proposes the correct Miller index of each spot in $\mathcal{Q}^*$. So to refine $\mathcal{A}$ we use these proposed indices and robust optimization. We use LTS with residual threshold annealing (as described in the main body of the paper) to determine the outliers of

$\mathcal{Q}$ and find the refinement of vector $\boldsymbol{a}$ that satisfies the most the Laue condition with target $h_{\boldsymbol{a},\boldsymbol{q}}$, i.e., we optimize the following expression.

$$\min_{\boldsymbol{w}\in\mathbb{R}^3} L_{\boldsymbol{a}} := \sum_{\boldsymbol{q}\in\mathcal{Q}^*} \left\| \boldsymbol{w}^\top \boldsymbol{q} - h_{\boldsymbol{a},\boldsymbol{q}} \right\|^2 .$$

Solving this optimization for each $\boldsymbol{a} \in \mathcal{A}$ yields a set of refined candidate vectors $\overline{\mathcal{A}} = \{\arg\min_{\boldsymbol{w}\in\mathbb{R}^3} L_{\boldsymbol{a}} : \boldsymbol{a} \in A\}$.

Recall that we know the ideal crystal lattice basis $\boldsymbol{M}_0^\star := (\mathbf{a}^\star, \mathbf{b}^\star, \mathbf{c}^\star)$. We now attach copies of $\boldsymbol{M}_0^\star$ to each of candidates vector of $\overline{\mathcal{A}}$ by aligning their first vector as follows. For each $\boldsymbol{a} \in \overline{\mathcal{A}}$, let $R_{\boldsymbol{a}}$ be any fixed rotation such that $R_{\boldsymbol{a}}\mathbf{a}^\star = \boldsymbol{a}$, i.e., a rotation that aligns $\mathbf{a}^\star$ with $\boldsymbol{a}$. For a given integer parameter r, we define the Rodrigues rotation matrix $R(i, r, \boldsymbol{a})$ that has $\boldsymbol{a}$ as eigenvector and that rotates $\frac{i\cdot 360}{r}$ degrees around the axis defined by $\boldsymbol{a}$. For each $\boldsymbol{a} \in \overline{\mathcal{A}}$, let $C_{\boldsymbol{a}} = \{R_{\boldsymbol{a}}R(i, r, \boldsymbol{a})\boldsymbol{M}_0^\star : i \in \{0, 1, \ldots, r-1\}\}$ be the set of r candidate basis, being rotated copies of $\boldsymbol{M}_0^\star$, associated with the vector $\boldsymbol{a}$. Doing this for all vectors of $\overline{\mathcal{A}}$ leads to a set

$$\mathcal{C} := \bigcup_{\boldsymbol{a}\in\overline{\mathcal{A}}} \mathcal{C}_{\boldsymbol{a}},$$

of rt 3×3 matrices, all being rotations of the given ideal basis $\boldsymbol{M}_0^\star$.

As a next step, we rank these candidate bases and take again the top t of them. To this end, for each $\boldsymbol{M} \in \mathcal{C}$ we define the score function

$$\textsc{score}(\boldsymbol{M}) := \sum_{\boldsymbol{q}\in\mathcal{Q}} \phi_\delta\left(\boldsymbol{M}\boldsymbol{q}\right),$$

and let $\mathcal{M}$ be the subset of $\mathcal{C}$ containing the top t basis according to their value $\textsc{score}(\boldsymbol{M})$. As mentioned above, each $\boldsymbol{M} \in \mathcal{M}$ induces a the set

$$\mathcal{V}_{\boldsymbol{q}}(\boldsymbol{M}) := \{\boldsymbol{v}_q := \textsc{round}(\boldsymbol{M}\boldsymbol{q}) : \boldsymbol{q} \in \mathcal{Q}f\} \subset \mathbb{Z}^3,$$

of possible Miller indices for $\mathcal{Q}$ that we can used as targets for our robust optimization.

That is, we use again the robust optimization described in equation (2) of the main body of the paper for each $\boldsymbol{M} \in \mathcal{M}$ using $\mathcal{V}_{\boldsymbol{q}}(\boldsymbol{M})$ as the target Miller indices.

In total, we have rt instances of the simplified problem that we solve in parallel and output the best solution. Notice that in principle, our approach is capable of handling multi-crystals, as solutions for each of the grains can be independently found in different instances.

As the size of our sampling increases, so does the chance of having a matrix $\boldsymbol{M} \in \mathcal{C}$ that is close to the indexing solution of our problem. In this case, the Miller indices induced by this sample will provide the desired oracle input for our robust optimization algorithms.

References

González, Á. (2010). *Mathematical Geosciences*, **42**, 49–64.

Paszke, A., Gross, S., Massa, F., Lerer, A., Bradbury, J., Chanan, G., Killeen, T., Lin, Z., Gimelshein, N., Antiga, L. *et al.* (2019). *Advances in neural information processing systems*, **32**.
